# Novel *KCNK3* variant in a child with pulmonary arterial hypertension

**DOI:** 10.1186/s41065-026-00680-z

**Published:** 2026-04-18

**Authors:** Yi-ming Zheng, Jia-qi Jiang, Xuan Li, Hong-biao Huang, Wen-yu Zhuo, Xuan Tang, Ying Liu, Hai-tao Lv

**Affiliations:** 1 Department of Pediatrics, Institute of Pediatric Research, Children’s Hospital of Soochow University, No. 92, Zhongnan Street, Suzhou Industrial Park, Suzhou, Jiangsu 215000 China; 2 Department of Cardiology, Children’s Hospital of Soochow University, No. 92, Zhongnan Street, Suzhou Industrial Park, Suzhou, Jiangsu 215000 China; 3 Department of Pediatrics, Fujian Provincial Hospital, Fujian Provincial Clinical College of Fujian Medical University, Fuzhou, 350000 Fujian China; 4 Department of Neonatology, People’s Hospital of Qianxinan Buyi and Miao Minority Autonomous Prefecture, Qianxinan, 562400 Guizhou China; 5 Department of Neonatology, Jiangyin People’s Hospital, Wuxi, 214000 Jiangsu China

**Keywords:** Apoptosis, KCNK3, NFE2L2, Oxidative stress, Pulmonary artery hypertension, Variation

## Abstract

**Background:**

Pathogenic variants in *KCNK3* have been implicated in pulmonary arterial hypertension (PAH); however, the molecular mechanisms underlying this association remain insufficiently defined.

**Methods:**

Whole-exome sequencing was performed in a child with PAH and her mother. The impact of the identified variant on protein stability was evaluated using cycloheximide chase assays. Apoptotic activity in transfected cells was assessed through flow cytometry and western blotting analysis. RNA sequencing was conducted to identify signaling pathways associated with altered gene expression. Oxidative stress levels were examined using inverted fluorescence microscopy. Expression levels of NFE2L2 was quantified by quantitative real-time polymerase chain reaction and western blotting.

**Results:**

A novel heterozygous *KCNK3* variant (c.607G > C, p.G203R) was identified. The substituted glycine residue demonstrated high evolutionarily conservation, and in silico analysis predicted structural alteration of the protein. The p.G203R variant was associated with reduced KCNK3 protein stability and an increase in apoptosis in vitro. Transcriptomic analysis indicated enhanced vascular smooth muscle cell migratory potential in cells expressing the variant. Increased cellular OS and apoptosis were observed in cells expressing p.G203R *KCNK3*. Bioinformatic analysis identified NFE2L2 as a key downstream effector. Expression of NFE2L2 was reduced in pulmonary artery endothelial cells expressing p.G203R *KCNK3*, while overexpression of NFE2L2 partially reversed variant-induced apoptosis.

**Conclusion:**

This study identifies a novel *KCNK3* p.G203R variant associated with PAH and provides mechanistic evidence supporting its pathogenicity. These findings expand the variant landscape of *KCNK3* in PAH and offer insights into disease pathogenesis that may inform future targeted therapeutic approaches.

**Supplementary Information:**

The online version contains supplementary material available at 10.1186/s41065-026-00680-z.

## Introduction

Pulmonary arterial hypertension (PAH) is a rare and multifactorial disorder influenced by both genetic predisposition and environmental exposures [1, 2]. Current therapeutic strategies primarily include pharmacological intervention and lung transplantation. Environmental contributors to PAH include medications, toxins, viral infections, inflammation, and hypoxia, whereas genetic factors predominantly involve pathogenic variants in specific genes. To date, eight genes have been implicated in the pathogenesis of PAH, including *TBX4*,* BMPR2*,* ENG*,* ACVRL1*,* KCNK3*,* SMAD9*,* EIF2AK4*, and *CAV1* [1, 3–11]. Among these, *BMPR2*,* ACVRL1*,* ENG*, and *CAV1* encode cell membrane proteins involved in angiogenesis [7, 10]. *TBX4* and *SMAD9* encode nuclear transcription factors, while *KCNK3* encodes the subunit of an ion channel located on the cell membrane [4, 10, 11]. *EIF2AK4* is involved in the regulation of protein phosphorylation and immune response [8].

The pathogenesis of PAH is characterized by excessive proliferation of pulmonary artery smooth muscle cells and dysfunction of pulmonary arterial endothelial cells [12]. Genetic evidence has indicated that endothelial cells are the primary initiating site in PAH, and endothelial apoptosis has been considered a pivotal early event in both experimental models and clinical contexts [12]. Additionally, impaired redox signaling within the pulmonary vasculature plays a central role in disease progression [13]. Excess production of reactive oxygen species (ROS) contributes to endothelial apoptosis and disruption of endothelial integrity.

This study focused on *KCNK3* (potassium channel subfamily K, member 3), in which a pathogenic variant was identified in a pediatric patient diagnosed with PAH. *KCNK3*, also known as *TASK-1* (TWIK-related acid-sensitive K+ channel), belongs to the two-pore-domain potassium (K2P) channel family, which is responsible for maintaining resting membrane potential [14, 15]. Each K2P channel subunit contains two pore-forming domains and four additional transmembrane segments. *KCNK3* functions as a pulmonary hypertension (pH)-sensitive potassium channel expressed in various tissues, including pulmonary arterial smooth muscle cells, endothelial cells, cardiomyocytes of the left and right atria and the right ventricle, adrenal glands, adipose tissue, and the central nervous system [16–22]. *KCNK3* has also been associated with primary aldosteronism, thermogenesis, and regulation of the blood–brain barrier [20–22]. In 2013, *KCNK3* was first identified as a PAH-associated gene in a familial case conducted by a genetic research consortium [11]. Since then, 12 distinct *KCNK3* variants have been reported in patients with PAH [23]. Given the role of potassium channels in regulating contraction, proliferation, survival, cell volume, and apoptosis of pulmonary vascular cells, disruptions in *KCNK3* function are considered mechanistically relevant to PAH pathogenesis [24].

In this study, a novel heterozygous *KCNK3* variant, c.607G > C, was identified, and in vitro experiments were conducted to assess its functional impact.

## Materials and methods

### Participants

A female pediatric patient diagnosed with PAH and her mother were enrolled from the Children’s Hospital of Soochow University, Suzhou, China. A comprehensive physical examination was conducted on the proband. Ethical approval for this study was granted by the Ethics Committee of the Children’s Hospital of Soochow University (No. 2021CS106), and the principles of the Declaration of Helsinki were followed. Informed consent was obtained from the mother.

Exome sequencing and variant identification: Peripheral blood samples (2 mL each) were collected from the child and her mother. Genomic DNA (gDNA) was extracted, and whole-exome sequencing was performed as previously described [24]. The mean sequencing depth across the target exome regions was 170.02×, with 98.85% of exonic regions covered at a minimum depth of 10×. Identified variants were confirmed by Sanger sequencing. Polymerase chain reaction (PCR) was used to amplify the genomic region containing the candidate variant, following established protocols [25]. The primer sequences used for amplification were as follows: forward 5′-GGAGGGAGAAGAAGGTTTCTG-3′ and reverse 5′-GTAGTGTGCGCGCTGCC-3′. Reverse primers were used to sequence the PCR products.

### Cell culture

Human pulmonary artery endothelial cells (hPAECs) were obtained from the American Type Culture Collection (ATCC, Manassas, VA, USA) and maintained in RPMI-1640 medium supplemented with 1% penicillin–streptomycin and 10% fetal bovine serum. Cultures were maintained at 37 °C in a humidified atmosphere containing 5% CO_2_. Cells were seeded at a density of 3.5 × 10^5^/mL. After 24 h, transfection with plasmids was performed using JetPrime (Polyplus) according to the instructions provided by the manufacturer.

### Plasmid construction

Complementary DNA (cDNA) sequences corresponding to the wild-type (WT) and p.G203R (*G203R*) variant of *KCNK3* and *NFE2L2*, were synthesized and subcloned into 3×FLAG-CMV-10 and pEGFP-N1 expression vectors, respectively. The integrity and sequence accuracy of the *KCNK3* inserts was confirmed by Sanger sequencing.

### Gene expression analysis

Plasmid DNA (1.5 µg per well) was transfected into hPAECs cultured in 12-well plates and incubated for 24 h. Cells were then washed with phosphate-buffered saline (PBS) and lysed in radioimmunoprecipitation assay (RIPA) buffer. Protein expression was analyzed by western blotting (WB) using a mouse monoclonal anti-FLAG antibody (66008-4-Ig, Proteintech) and anti-β-actin antibody (41185s, CST). Band intensities were quantified using Gel-Pro Analyzer 4 (Media Cybernetics, L.P.).

### Protein stability assay for WT and *KCNK3* variant

To evaluate protein stability, hPAECs were transfected with 1.5 µg of either WT or G203R KCNK3 plasmids in 12-well plates. After 24 h, cells were treated with cycloheximide (CHX; 25 µg/mL, HY-12320, MedChemExpress) for 0, 1, 2, 3, or 4 h. Cell lysates were then collected and analyzed byWB.

### Annexin V apoptosis assay

Flow cytometry was used to assess early and late apoptosis. A total of 3.5 × 10⁵ hPAECs per well were seeded in six-well plates and transfected as described. At 24 h post-transfection, cells were harvested and processed using an Annexin V/propidium iodide (PI) apoptosis detection kit (BD Biosciences, Heidelberg, Germany), following the manufacturer’s protocol. Cells were centrifuged at 1000 rpm for 3 min, washed twice with PBS at 4 °C, and resuspended in 400 µL binding buffer. Annexin V and PI staining was performed for 15 min in the dark. The proportion of apoptotic cells was quantified using flow cytometry (BD Biosciences, Heidelberg, Germany).

### Intracellular ROS and Oxidative Stress (OS) assessment

Intracellular ROS levels were measured using dihydroethidium (DHE) staining. At 24 h post-transfection, hPAECs were incubated with DHE (Molecular Probes) in the dark at 37 °C for 15 min. Following DHE staining, cells were incubated with Hoechst (1:1000 dilution) at 37 °C for 5 min in the dark. Fluorescence signals were examined using an inverted fluorescence microscope with an excitation wavelength of 488 nm and emission detection at 530 nm.

### Quantitative real-time PCR

Total RNA was extracted from transfected hPAECs using TRIzol reagent (15996–026, Invitrogen, USA). First-strand cDNA was synthesized using the PrimeScript RT reagent kit (RR047A, Takara Bio, China). Quantitative real-time PCR (qRT-PCR) was carried out using SYBR Green detection chemistry (FP205, Tiagen, China) on a CFX384 Touch™ Real-Time PCR Detection System. Each reaction was conducted in triplicate. β-actin was used as the reference control. Primer sequences used for qRT-PCR are provided in Table S1.

## Results

### Clinical manifestations

A female pediatric patient aged 12 years and 10 months was admitted to the hospital following an episode of syncope that occurred 3 h prior to presentation. The episode was characterized by upward deviation of the eyes, cyanosis of the lips, a bluish facial complexion, and loss of consciousness, which resolved spontaneously after approximately one minute. Following recovery, she reported experiencing headache, transient loss of sensation and motor function in both lower limbs, and a single episode of vomiting of gastric contents. Her medical history indicated normal physical development milestones and an unremarkable perinatal history. The mother reported no abnormalities during her own birth or the patient’s delivery. The father was reported to have died from a reported “stomach perforation and gastric ulcer.”

**Fig. 1:**
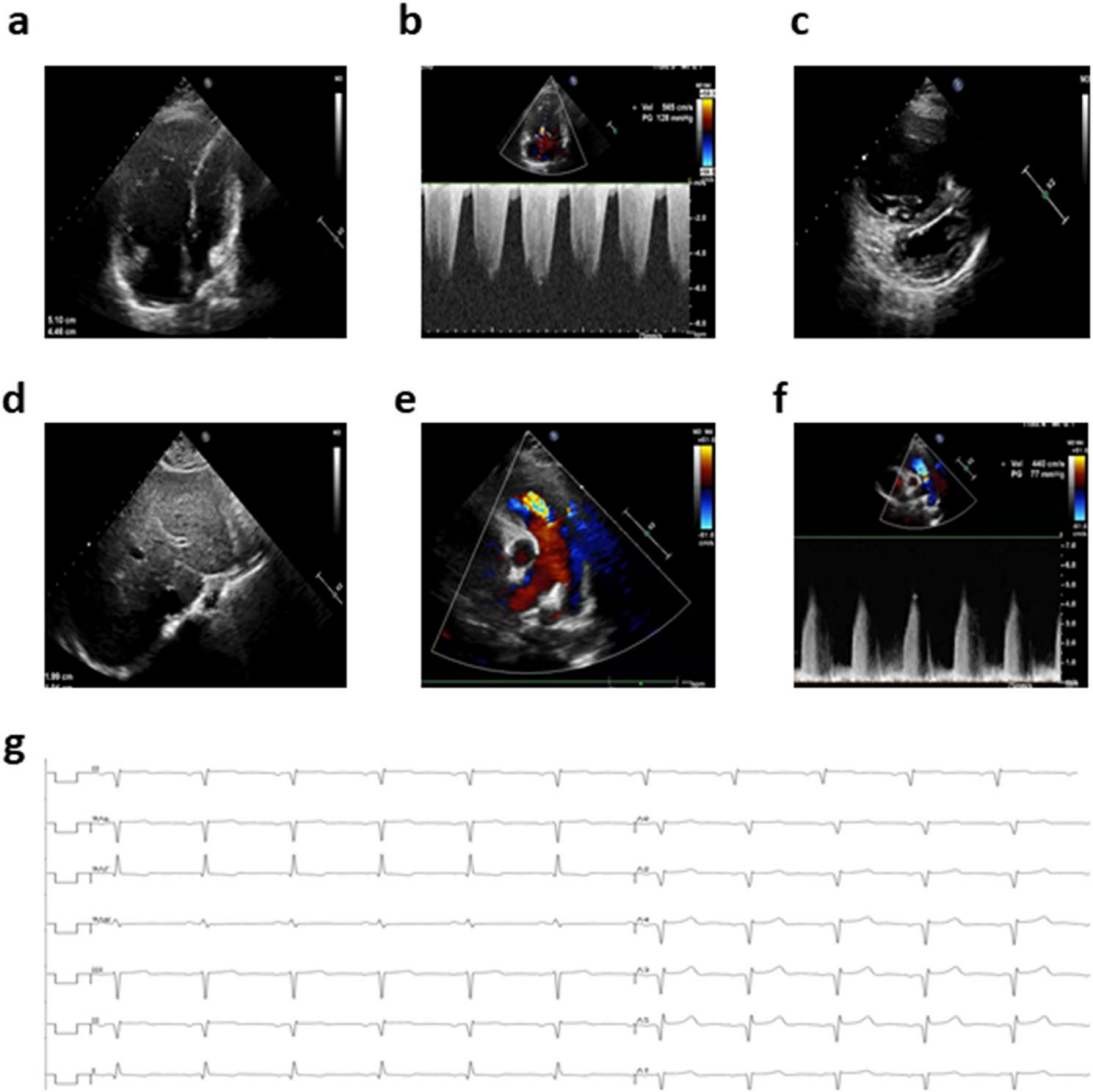
Echocardiographic and electrocardiographic characteristics of PAH. **a** Apical four-chamber views presenting significant enlargement of the right atrium (RA) and right ventricle (RV). **b** Doppler tracing of tricuspid regurgitation (TR) demonstrating severe pulmonary hypertension (TRV > 5 m/s). **c** Parasternal short-axis views indicating interventricular septal (IVS) displacement towards the left ventricle (LV) and RV enlargement. **d** Subcostal view showing dilatation of the inferior vena cava (IVC). **e**–**f** Pulmonary regurgitation (PR) Doppler tracings presenting increased regurgitant velocity (PRV > 4 m/s). **g** ECG demonstrating sinus rhythm, RV enlargement, prolonged QT interval, and ST–T segment changes. Abbreviations: RA, right atrium; RV, right ventricle; TR, tricuspid regurgitation; PR, pulmonary regurgitation; PH, pulmonary hypertension

At the time of admission, the patient’s weight was 62 kg (2–3 SD), height was 156 cm (-2 to -1 SD), and blood pressure was 150/94 mmHg. Transthoracic echocardiography demonstrated enlargement of the right atrium and right ventricle, right ventricular wall hypertrophy, and significantly elevated pulmonary arterial pressure consistent with a diagnosis of severe PAH (Fig. 1a–f). Electrocardiography (ECG) demonstrated right ventricular enlargement, which is characteristic of PAH (Fig. 1g).

Myocardial enzyme levels were as follows: CK 107.9 U/L (reference range: 25–225), CK-MB 2.6 ng/mL (0–3.61), and NT-proBNP 212.6 pg/mL (0.0–125.0). Routine blood tests, liver and kidney function tests, and immunological assays presented no abnormalities.

### Identification of a novel missense variant in *KCNK3*

**Fig. 2:**
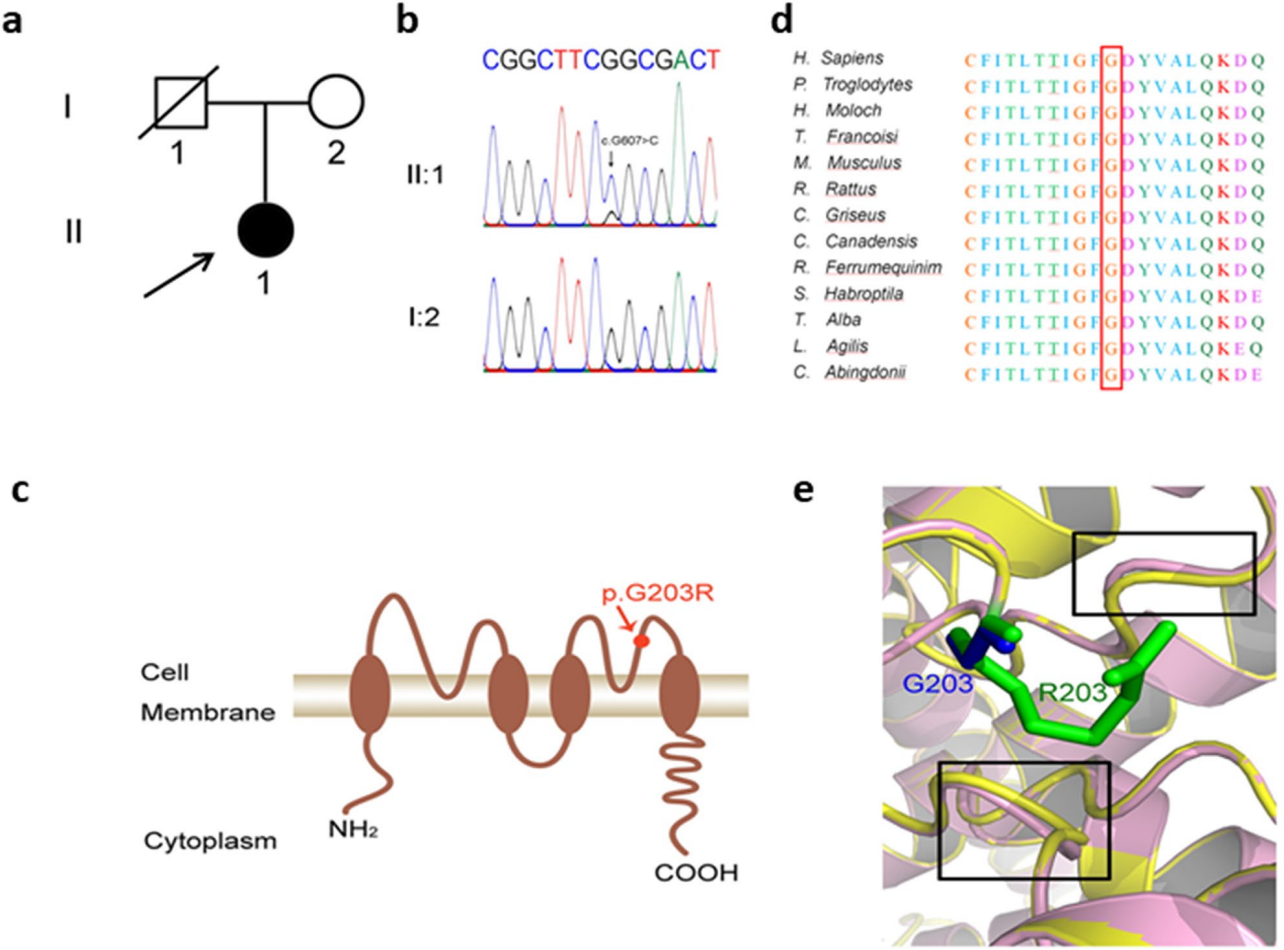
Genetic analysis of the *KCNK3* variant (**a**) Pedigree of the family; the proband is indicated by an arrow. **b** Sanger sequencing of the region containing the variant. **c** Schematic structure of the *KCNK3* protein, with the variant site marked by an arrow. **d** Multiple sequence alignment showing evolutionary conservation of residue G203 across species. **e** Predicted 3D structural changes of the wild-type and variant proteins. R203 residue is depicted in green, wild-type G203 in blue, wild-type peptide in pink, and the variant peptide in yellow

Peripheral blood samples were collected from the patient (II:1) and her healthy mother (I:2) (Fig. 2a), followed by genomic DNA extraction and whole-exome sequencing. A heterozygous missense variant, c.607G > C in *KCNK3* (NM_002246), was identified in the patient, while the mother carried the wild-type allele. The presence of the *KCNK3* variant was confirmed by Sanger sequencing (Fig. 2b). This variant was absent from both the ExAC and 1000 Genomes databases. The c.607G > C variant resulted in a substitution of glycine (G) with arginine (R) at codon 203 located within the second pore-forming domain of the KCNK3-encoded potassium channel protein (Fig. 2c). Cross-species sequence alignment demonstrated that this residue is highly conserved (Fig. 2d), indicative of functional importance. According to MutationTaster (https://www.mutationtaster.org/), the p.G203R substitution was classified as disease-causing. Three-dimensional structural models of the wild-type and variant proteins were constructed using SWISS-MODEL (https://swissmodel.expasy.org/), and hydrogen bonding was analyzed using PyMOL (Fig. 2e). The molecular structure of the variant-containing protein was longer than that of the wild type, which may have altered protein conformation and affected its activity. Based on these results, and following the criteria of the American College of Medical Genetics and Genomics, the variant was classified as likely pathogenic. Further investigations were conducted to examine the underlying pathogenic mechanisms.

### The p.G203R variant reduces KCNK3 protein stability and induces apoptosis in vitro

**Fig. 3:**
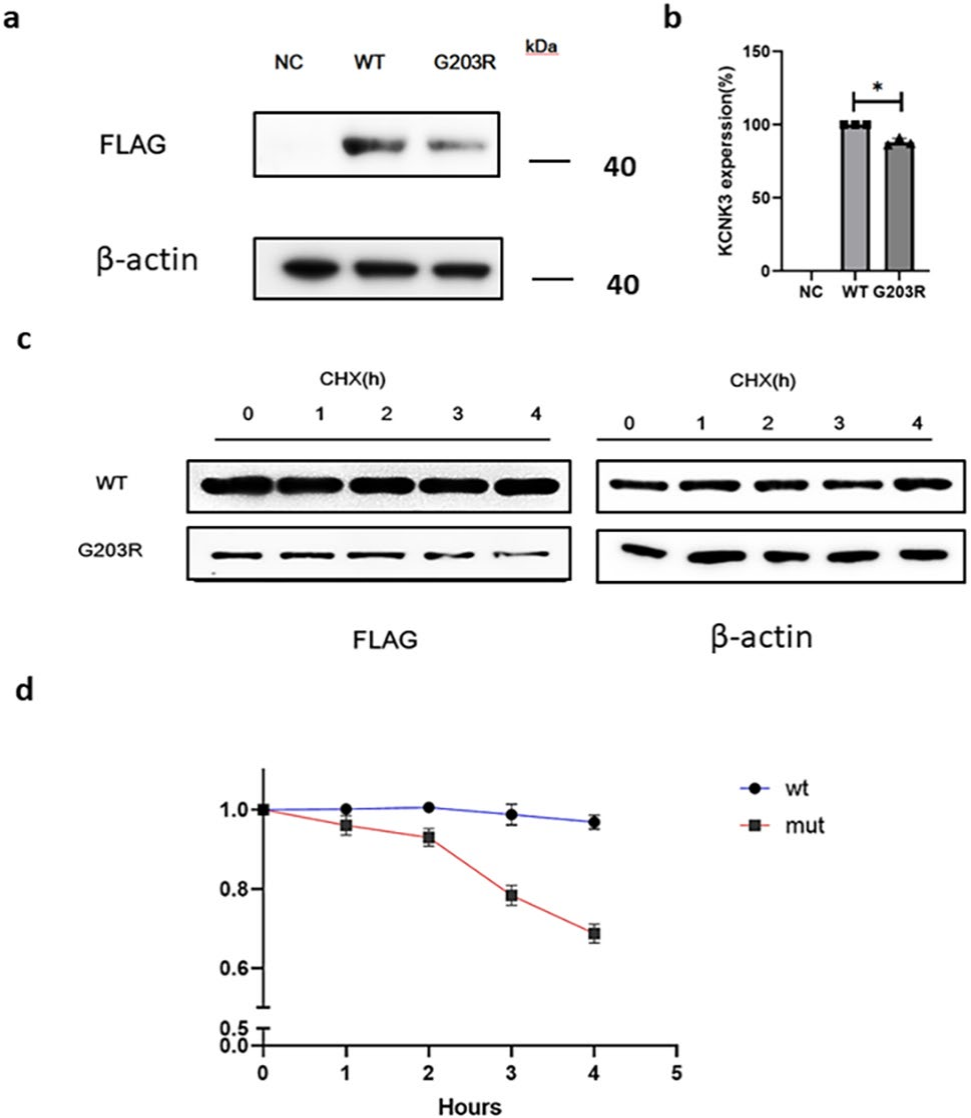
The p.G203R *KCNK3* variant reduces protein stability and induces apoptosis in vitro (**a**) Western blot analysis presenting reduced expression of p.G203R KCNK3 compared with wild type (WT). **b** Quantification of FLAG-tagged WT and p.G203R KCNK3 expression using Gel-Pro Analyzer. **c** Cycloheximide (CHX) chase assay depicting reduced stability of the p.G203R KCNK3 protein. **d** Quantification of protein degradation over 0, 1, 2, 3, and 4 h post-CHX treatment. **e**–**f** Flow cytometry analysis of apoptosis in hPAECs transfected with WT or p.G203R KCNK3 plasmids for 24 h. The FLAG group served as the negative control. *p < 0.05, **p < 0.001; data are presented as mean ± SD and analyzed using t-tests

Given the predicted pathogenic potential of the p.G203R *KCNK3* variant, in vitro studies were conducted to evaluate its functional impact. hPAECs were transfected with FLAG-tagged plasmids encoding either wild-type or p.G203R *KCNK3* at equivalent concentrations. Protein expression was assessed using WB (Fig. 3a). The p.G203R variant resulted in significantly reduced KCNK3 protein levels compared to the WT control (Fig. 3b). Cells were then treated with cycloheximide, an inhibitor of protein synthesis, to determine if reduced protein levels were due to degradation [26]. The results indicated that p.G203R *KCNK3* exhibited reduced protein stability when compared to the wild-type protein (Fig. 3c–d). These findings suggest that the p.G203R substitution compromises KCNK3 protein integrity, potentially impairing its physiological function and contributing to disease pathogenesis.

### RNA-seq indicates dysregulated pathways in cells expressing p.G203R *KCNK3*

**Fig. 4:**
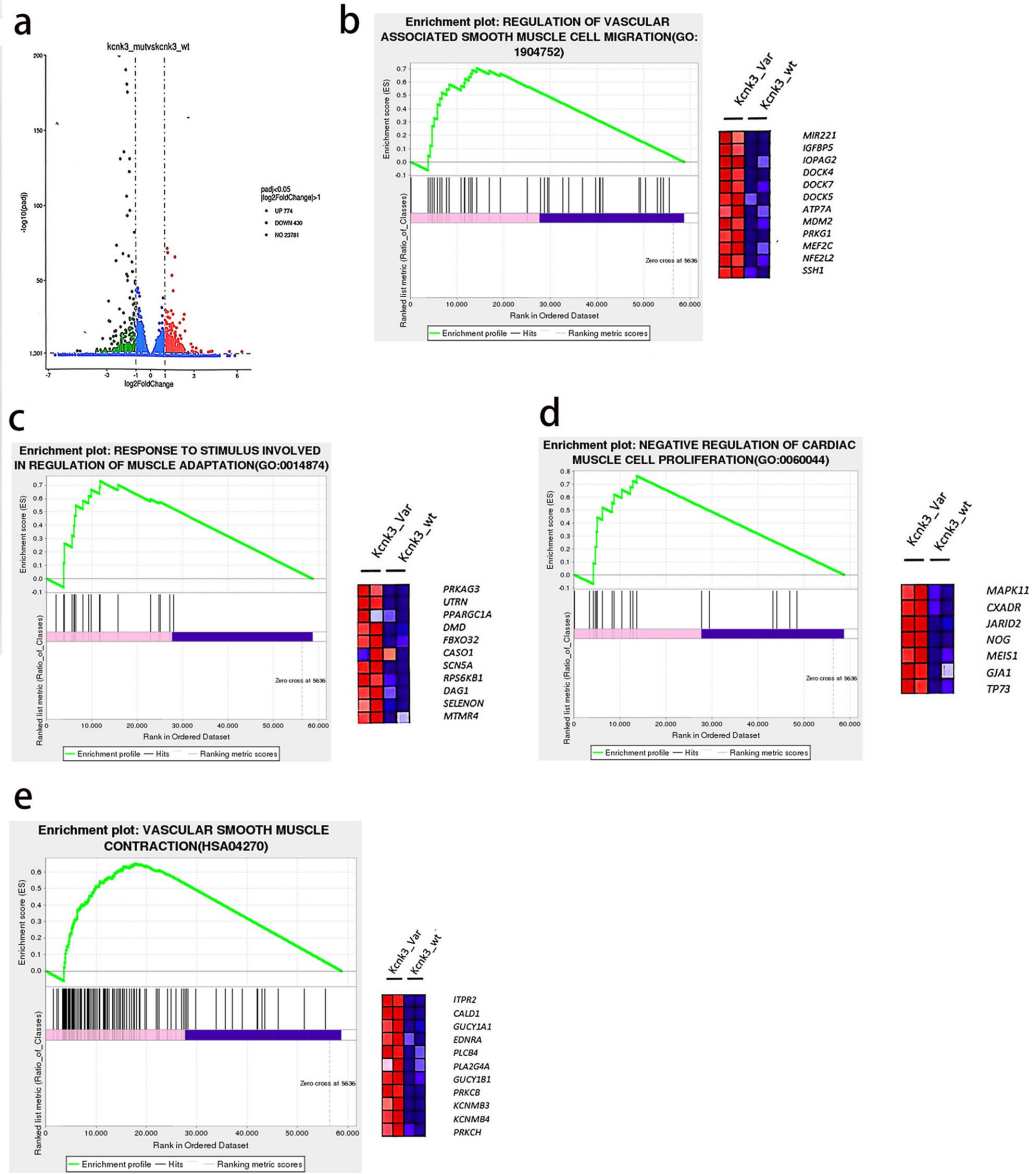
RNA Sequencing Analysis of Pathways Affected by p.G203R *KCNK3. * (**a**) Volcano plot of differentially expressed genes showing 774 upregulated and 430 downregulated genes in p.G203R-expressing cells compared with WT. **b**–**e** KEGG pathway analysis presenting positive association with regulation of vascular smooth muscle cell (VSMC) migration and muscle adaptation, and negative association with cardiac muscle cell proliferation and vascular smooth muscle contraction

RNA sequencing (RNA-seq) was conducted to examine the molecular pathways affected by the p.G203R variant. hPAECs were transfected with either wild-type or p.G203R *KCNK3* plasmids and cultured for 48 h prior to RNA extraction. RNA-seq data indicated that 430 genes were downregulated and 774 were upregulated in cells expressing the p.G203R variant compared with the wild type (Fig. 4a). Kyoto Encyclopedia of Genes and Genomes (KEGG) enrichment analysis demonstrated that p.G203R *KCNK3* was positively associated with the regulation of vascular smooth muscle cell (VSMC) migration (Fig. 4b) and responses to stimuli involved in muscle adaptation (Fig. 4c). In contrast, negatively associations were observed with pathways regulating cardiac muscle cell proliferation (Fig. 4d) and vascular smooth muscle contraction (Fig. 4e). These pathways are closely linked to the pathogenesis and pathophysiological changes observed in pulmonary hypertension. The findings support a role for the p.G203R *KCNK3* variant in PAH development and validate the functional significance of the observed alterations in gene expression.

### The p.G203R variant is associated with enhanced apoptosis

**Fig. 5:**
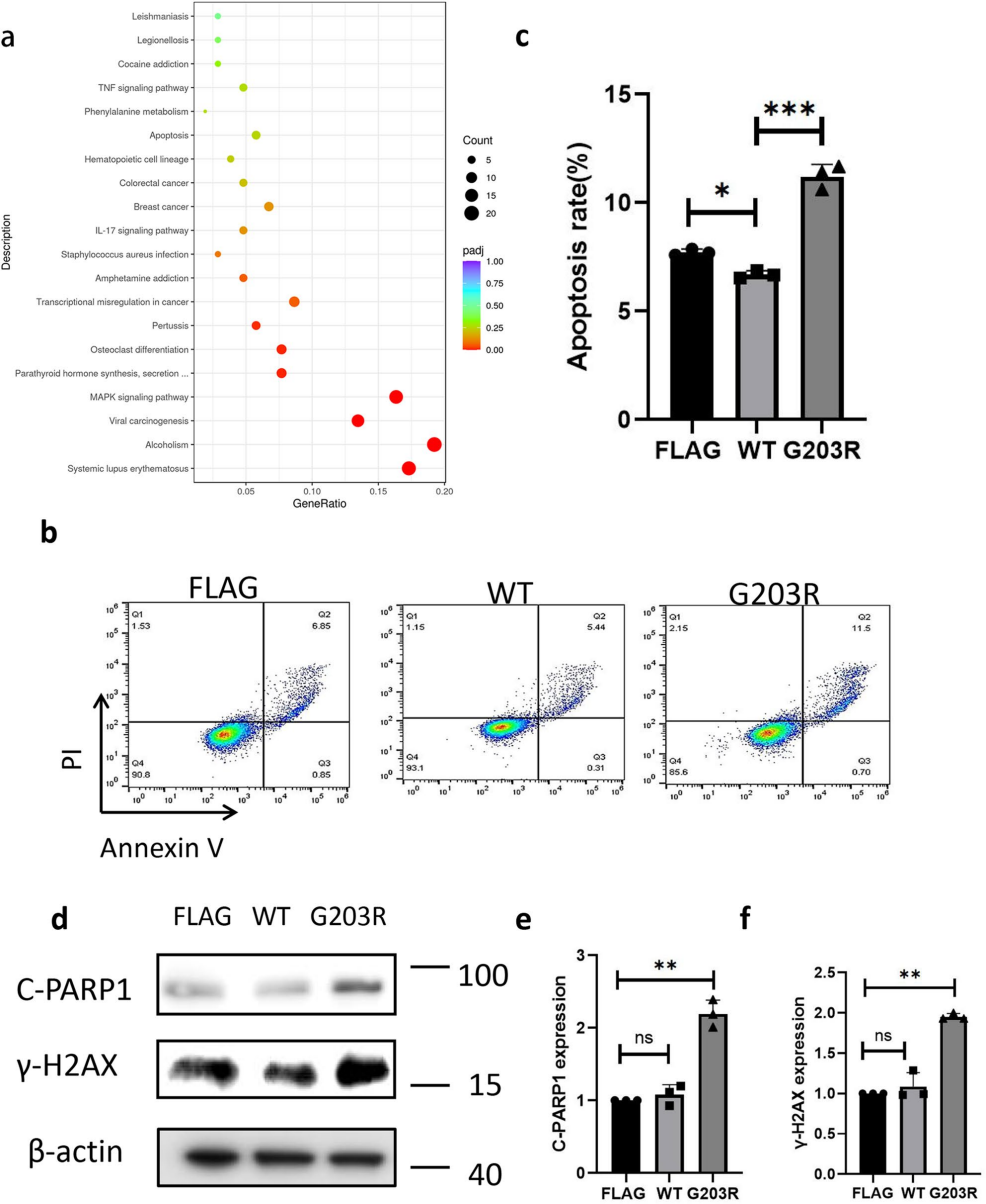
p.G203R *KCNK3* variant induces apoptosis in hPAECs.(**a**) KEGG pathway enrichment indicating involvement in apoptosis-related signaling. **b**–**c** Annexin V-FITC/PI staining after transfection with WT or c.607G > C (KCNK3) plasmids for 24 h demonstrating increased apoptosis. **d** Western blot analysis demonstrating elevated γ-H2AX and cleaved PARP1 levels in the p.G203R variant group compared with the FLAG control. **e**–**f** Densitometric analysis showing increased γ-H2AX and cleaved PARP1 expression in the p.G203R group. (ns *p* > 0.05; **p < 0.01)

Transcriptomic analysis the p.G203R *KCNK3* variant was linked to the dysregulation of apoptosis-related pathways (Fig. 5a). At 48 h post-transfection, hPAECs expressing the p.G203R variant demonstrated reduced cell coverage compared with the wild-type group (Figure S1). Flow cytometry analysis indicated significantly increased apoptosis in hPAECs transfected with the p.G203R *KCNK3* plasmid (Fig. 5b–c). Additionally, WB analysis was conducted to examine the expression of apoptosis-related marker proteins γ-H2AX and cleaved PARP-1. Increased expression of γ-H2AX and cleaved PARP-1 was observed in the *KCNK3* c.607G > C variant group compared with the wild type (Fig. 5d–f), indicating that the variant promoted apoptosis.

### OS induced by the*KCNK3*c.607G > C variant in hPAECs and its potential role in PAH pathogenesis

**Fig. 6:**
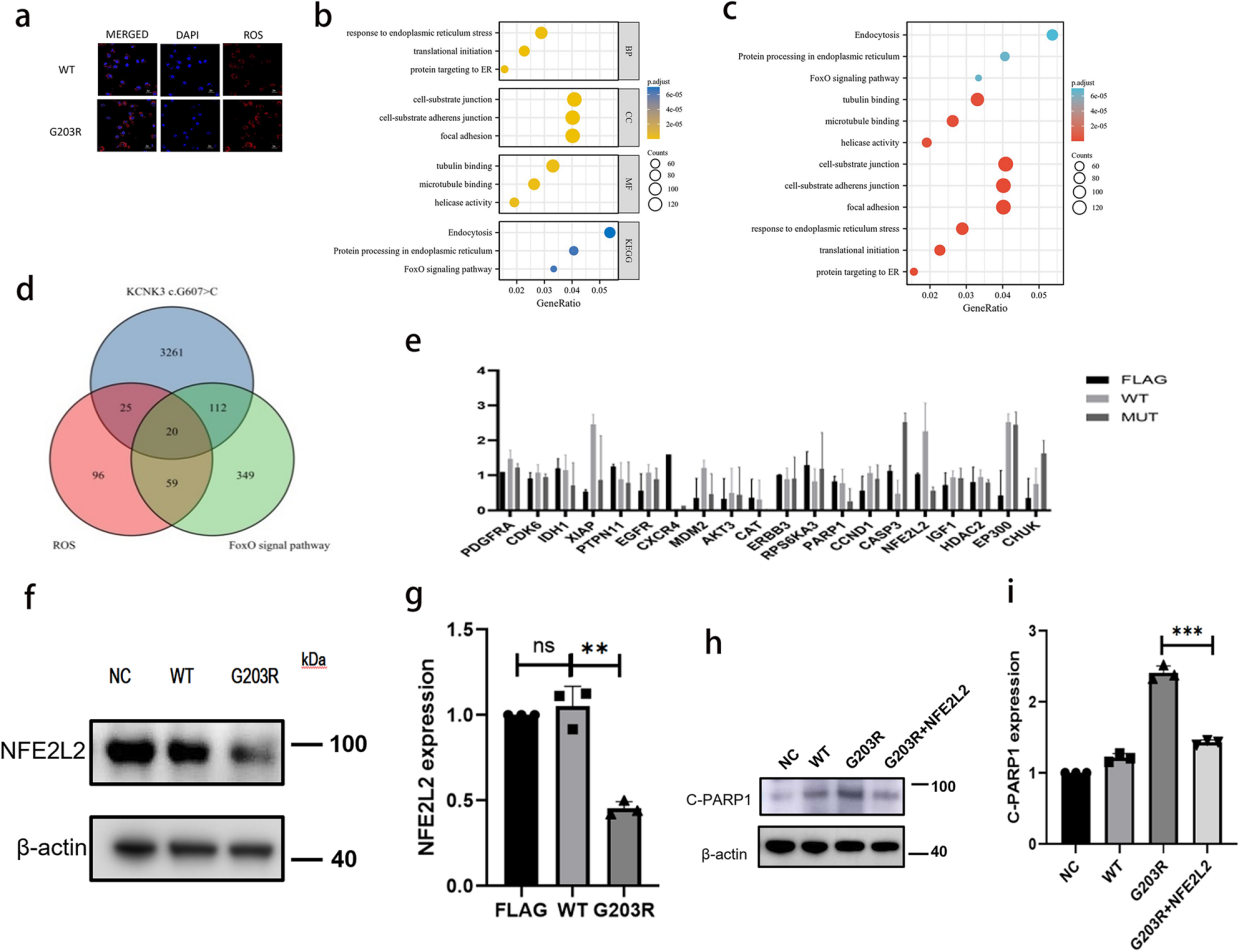
The p.G203R *KCNK3* variant induces oxidative stress and downregulates NFE2L2. **a** Immunofluorescence showing increased ROS levels in hPAECs transfected with KCNK3 c.607G > C plasmid. **b**–**c** GO and KEGG pathway enrichment analyses highlighting altered expression of genes involved in oxidative stress and FOXO signaling. **d** Venn diagram identifying 20 overlapping differentially expressed genes involved in FOXO signaling and oxidative stress. **e** qRT-PCR validation of the 20 genes; NFE2L2 was identified as significantly altered. **f**–**g** Western blot and densitometry analysis conforming reduced NFE2L2 protein expression in the p.G203R KCNK3 group. **h**–**i** Co-transfection of NFE2L2 plasmid partially reversed apoptosis, as indicated by reduced cleaved PARP1 levels compared to cells transfected with p.G203R KCNK3 alone. (ns *p* > 0.05; **p < 0.01; ***p < 0.001)

OS has been reported to be associated with PAH, intracellular OS levels were assessed using immunofluorescence [27]. Increased OS levels were observed in hPAECs transfected with the *KCNK3* c.607G > C plasmid compared with those transfected with the wild-type plasmid (Fig. 6a). RNA sequencing analysis also indicated enrichment of the Forkhead box O (FOXO) signaling pathway, which plays a key role in the regulation of OS (Fig. 6b–c).

Genes associated with OS and FOXO signaling were obtained from GeneCards (https://www.genecards.org/) and cross-referenced with the differentially expressed genes identified in the RNA-seq dataset. This analysis yielded 20 common differentially expressed genes (Fig. 6d): *PDGFRA*,* CDK6*,* IDH1*,* XIAP*,* PTPN11*,* EGFR*,* CXCR*,* MDM2*,* AKT3*,* CAT*,* ERBB3*,* RPS6KA3*,* PARP1*,* CCND1*,* CASP3*,* NFE2L2*,* IGF1R*,* HDAC2*,* EP300*,* and CHUK*. Expression of these genes was subsequently validated using PCR (Fig. 6e). Among them, *NFE2L2* (nuclear factor erythroid 2-like 2) was identified as a key transcription factor involved in the oxidative stress response, based on both PCR validation and supporting literature.

WB analysis further confirmed a reduction in NFE2L2 protein levels in hPAECs transfected with the *KCNK3* c.607G > C plasmid (Fig. 6f–g), consistent with transcript-level findings and bioinformatic predictions. To assess if *NFE2L2* could modulate apoptosis associated with this variant, co-transfection experiments were conducted using *NFE2L2* and *KCNK3* c.607G > C plasmids. Expression of cleaved PARP1, an apoptosis marker, was then evaluated by WB. The results (Fig. 6h–i) indicated increased cleaved PARP1 levels in cells co-transfected with *KCNK3* c.607G > C and *NFE2L2*, compared with cells transfected with the *KCNK3* variant alone, indicating that *NFE2L2* overexpression partially compensated for apoptosis induced by *KCNK3* c.607G > C.

## Discussion

According to the 2022 European Society of Cardiology (ESC) and European Respiratory Society (ERS) Guidelines for the Diagnosis and Treatment of PH, the condition is classified into five subtypes based on hemodynamic characteristics, pathological features, and clinical diagnostic modalities [2]. These subtypes include PAH, PH due to left heart disease, PH associated with lung disease or hypoxia, chronic thromboembolic PH, and PH with multifactorial or unclear mechanisms. Despite advances in classification and management, the pathogenesis of PAH continues to be a major focus of research.

In the present study, a novel heterozygous missense variant in *KCNK3* (c.607G > C; p.G203R), was identified in a pediatric patient diagnosed with PAH. This variant resulted in decreased KCNK3 protein expression, likely due to decreased protein stability. Transcriptomic profiling revealed significant alterations in gene expression, with 1,204 differentially expressed genes identified in hPAECs expressing the p.G203R variant. Pathway enrichment analysis indicated positive associations with the regulation of VSMC migration and response to stimuli involved in muscle adaptation, and negative associations with the regulation of cardiac muscle cell proliferation and VSMC contraction. These changes may contribute to key pathological features of PAH, including reduced vascular elasticity, vascular wall thickening and increased pulmonary arterial pressure. Excessive VSMC proliferation, enhanced cellular adaptability, reduced cardiomyocyte proliferation, and increased myocardial cell contraction have all been associated with PH [28–30].

These findings are consistent with prior evidence and further support the pathogenic potential of the KCNK3 c.607G > C variant in the context of PAH. Channels containing KCNK3 have been widely recognized to mediate background K⁺ currents that stabilize resting membrane potential [31]. In this study, the identified p.G203R variant is located within the conserved GXG motif of the second pore-domain selectivity filter a region critical for channel function. The variant resulted in decreased protein stability, which may have impaired channel activity and contributed to the development of PAH. However, due to experimental limitations, membrane potential measurements could not be conducted, and intracellular and extracellular ion levels were not assessed. The apparent paradox between KCNK3 variant-induced endothelial apoptosis and the hyperproliferative PAH phenotype may be reconciled by considering the “two-hit” hypothesis of PAH pathogenesis: initial endothelial injury and apoptosis create a pro-inflammatory microenvironment that subsequently drives compensatory proliferation of apoptosis-resistant endothelial clones and smooth muscle cells. Our findings align with prior evidence that endothelial apoptosis represents an early pathogenic event, whereas hyperproliferation emerges during later disease stages. The stage-specific effects of KCNK3 dysfunction—early endothelial loss followed by dysregulated vascular remodeling—warrant further investigation in longitudinal models.

Apoptosis of hPAECs may contribute to vascular endothelial dysfunction, a recognized pathological feature of PAH. In addition, vascular remodeling, which underlies the progression of PAH, is closely associated with apoptosis of endothelial and smooth muscle cells [32]. In this study, flow cytometry demonstrated that the p.G203R KCNK3 variant increased apoptosis, consistent with findings reported by Le Ribeuz et al. [33]. Dysregulation of redox signaling is involved in pathological processes such as hypertension and pulmonary vascular remodeling [33]. ROS play a central role in the pathogenesis of PAH and are known to drive redox signaling imbalances that promote endothelial dysfunction and vascular remodeling.

Bioinformatic analysis, supported by validation through qRT-PCR and WB, identified *NFE2L2* as a differentially expressed gene and protein. NFE2L2 is a key transcription factor expressed in various tissues and involved in enzyme-mediated antioxidant pathways in cardiomyocytes [34, 35]. NFE2L2 regulates cellular responses to oxidative stress, prevents oxidative damage, maintains redox homeostasis, and influences drug metabolism, antioxidant defense, proteasome function, cell proliferation, and metabolism [36, 37]. Additional studies have implicated NFE2L2 in the regulation of autophagy, apoptosis, and the activity of inflammasomes, and stem cell populations [38]. In hPAECs expressing the *KCNK3* p.G203R variant, NFE2L2 expression was reduced; therefore, it is hypothesized that reduced NFE2L2 expression contributes to increased oxidative stress and apoptosis in these cells. However, the precise molecular mechanisms by which *KCNK3* variants influence NFE2L2 signaling require further elucidation.

Current treatment strategies for PH include general supportive measures, pharmacological therapy, and surgical intervention [2]. General measures comprise physical activity guidance, anticoagulation, diuretics, and supplemental oxygen therapy. Pharmacological options include calcium channel blockers (nifedipine, diltiazem, amlodipine), endothelin receptor antagonists (ambrisentan, bosentan, macitentan), phosphodiesterase type 5 inhibitors and soluble guanylate cyclase stimulators (sildenafil, tadalafil, riociguat), prostacyclin analogues, and prostacyclin receptor agonists (epoprostenol, iloprost, treprostinil, beraprost). Surgical strategies include interventional procedures and lung or heart–lung transplantation. Currently, PAH therapy mainly relies on oral medications; however, existing treatments do not specifically target endothelial cell apoptosis or pathways involving KCNK3 and NFE2L2. These pathways represent potential directions for future therapeutic research. This study has several limitations. First, the genetic findings are based on a single patient without validation in additional unrelated cases or familial segregation analysis due to the unavailability of paternal genotype, limiting the generalizability of our results. Second, quantitative hemodynamic data, including precise pulmonary arterial pressure measurements and detailed echocardiographic parameters, were not included, which would have strengthened the clinical characterization of PAH severity. Third, the molecular mechanisms linking KCNK3 variants to NFE2L2 downregulation require further elucidation.

In summary, a novel heterozygous missense variant in *KCNK3* was identified in a pediatric patient with PAH. Functional studies indicated that this variant reduced protein stability, disrupted redox homeostasis, and promoted endothelial apoptosis—processes relevant to PAH pathophysiology. These findings not only provide new insights into the molecular mechanisms underlying PAH but may also inform future genetic counseling and the development of targeted therapies.

## Conclusion

Clinical evaluation and in vitro functional assays indicate that the *KCNK3* p.G203R variant may contribute to the pathogenesis of PAH by inducing endothelial cell apoptosis. The expression of p.G203R *KCNK3* in hPAECs was significantly reduced, likely due to decreased protein stability. Transcriptomic analysis of RNA-seq data indicated that this variant was associated with upregulation of pathways involved in VSMC migration and muscle adaptation, and downregulation of pathways regulating cardiac muscle cell proliferation and VSMC contraction, all of which are processes related to PAH pathogenesis. Additionally, the variant downregulated NFE2L2, leading to increased oxidative stress and apoptosis, suggesting a potential mechanism underlying PAH. This study expands the known variant spectrum of *KCNK3*, providing valuable data for genetic counseling. Future research should explore therapeutic strategies targeting this specific *KCNK3* variant, including small-molecule modulators aimed at restoring protein function or gene therapy approaches to re-establish normal channel expression. Further research into NFE2L2-mediated antioxidant pathways may also support the development of therapies that address oxidative stress and endothelial dysfunction in PAH. 

## Supplementary Information

Supplementary Material 1.

Supplementary Material 2.

## Data Availability

The datasets used or analysed during the current study are available from the corresponding author on reasonable request.

## Abbreviations

PAH: Pulmonary artery hypertension
OS: Oxidative stress
TBX4: T-Box Transcription Factor 4
BMPR2: Bone morphogenetic protein type 2 receptor
ENG: Endoglin
ACVRL1: Activin A receptor type II-like Kinase 1
SMAD9: SMAD Family Member 9
EIF2AK4: Eukaryotic Translation Initiation Factor 2 Alpha Kinase 4
CAV1: Caveolin 1
PASMCs: Pulmonary artery smooth muscle cells
PAEC: Pulmonary arterial endothelial cell
ROS: Reactive oxygen species
KCNK3: Potassium channel subfamily K, member 3
TASK-1: Twik-related-acid-sensitive-K+ channel
K2P: K+ conductance channel family or two-pore-domain
SMCs: Smooth muscle cells
ECs: Endothelial cells
gDNA: Genomic DNA
PCR: Polymerase Chain Reaction
PS: Penicillin-streptomycin
FBS: Fetal bovine serum
WT: Wild-type
NFE2L2: Nuclear factor derived 2-like 2
WB: Western blot
CHX: Cycloheximide
PI: Propidium iodide
DHE: Dihydroethidium
ECG: Electrocardiography
CKMB: Creatine Kinase-MB
NT-proBNP: N-terminal pro-B-type natriuretic peptide
WES: Whole-exome sequencing
KEGG: Kyoto Encyclopedia of Genes and Genomes
γ-H2AX: Gamma-H2A histone family member X
FOXO: Forkhead Box O
PARP-1: Poly (ADP-ribose) Polymerase 1
PDGFRA: Platelet-Derived Growth Factor Receptor Alpha
CDK6: Cyclin-Dependent Kinase 6
IDH1: Isocitrate Dehydrogenase 1
XIAP: X-Linked Inhibitor of Apoptosis Protein
PTPN11: Protein Tyrosine Phosphatase, Non-Receptor Type 11
EGFR: Epidermal Growth Factor Receptor
CXCR: C-X-C Chemokine Receptor
MDM2: Mouse Double Minute 2 Homolog
AKT3: AKT Serine/Threonine Kinase 3
CAT: Catalase
ERBB3: Erb-B2 Receptor Tyrosine Kinase 3
RPS6KA3: Ribosomal Protein S6 Kinase Alpha-3
PARP1: Poly(ADP-Ribose) Polymerase 1
CCND1: Cyclin D1
CASP3: Caspase 3
IGF1R: Insulin-Like Growth Factor 1 Receptor
HDAC2: Histone Deacetylase 2
EP300: E1A Binding Protein P300
CHUK.: Conserved Helix-Loop-Helix Ubiquitous Kinase
PH: Pulmonary hypertension
VSMC: Vascular smooth muscle cell
GXG: Where X refers to all amino acids
GSEA: Gene set enrichment analysis
