# Supplementary figures and images for: Novel KCNK3 variant in a child with pulmonary arterial hypertension
Source: Hereditas. 2026 Apr 18;163:67. doi: 10.1186/s41065-026-00680-z (PMC13224539; doi:10.1186/s41065-026-00680-z)

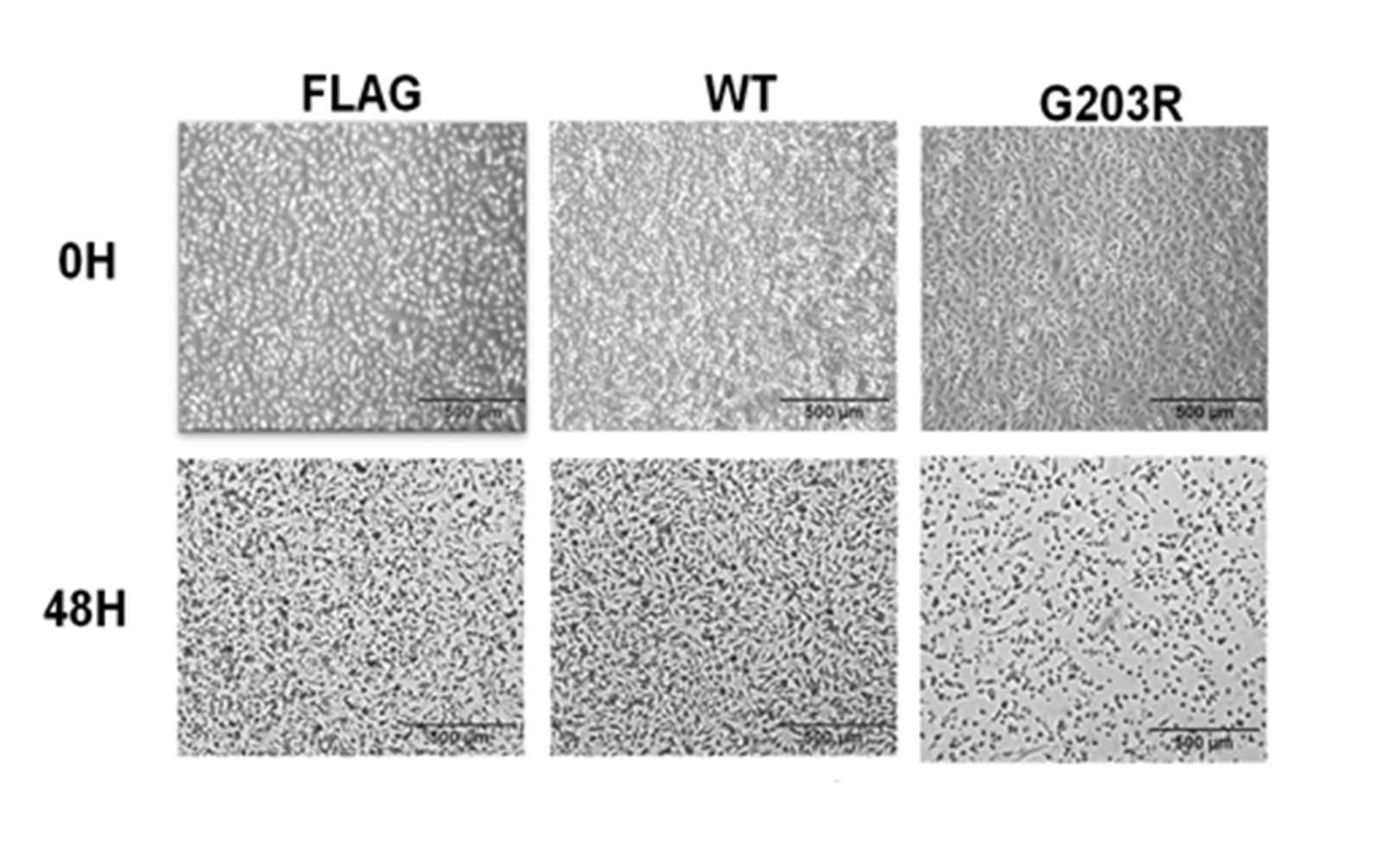

Supplement: Supplementary file 1 — Supplementary Material 1. [file 41065_2026_680_MOESM1_ESM.tif]
