# Supplementary material for: Novel KCNK3 variant in a child with pulmonary arterial hypertension
Source: Hereditas. 2026 Apr 18;163:67. doi: 10.1186/s41065-026-00680-z (PMC13224539; doi:10.1186/s41065-026-00680-z)
Supplement: Supplementary file 2 — Supplementary Material 2. [file 41065_2026_680_MOESM2_ESM.docx]

Table S1. The qRT-PCR primer sequences

| Gene name |  | Sequence(5’-3’) |
| --- | --- | --- |
| Human Actin | Forward primer | CCTTCCTGGGCATGGAGTC |
|  | Reverse primer | TGATCTTCATTGTGCTGGGTG |
| Human AKT3 | Forward primer | CAGAGGCAAGAAGAGGAGAGAAT |
|  | Reverse primer | GTGCCACTTCATCCTTTGCAATA |
| Human CASP3 | Forward primer | TGAGCCATGGTGAAGAAGGAATAA |
|  | Reverse primer | CCCGGGTAAGAATGTGCATAAAT |
| Human CAT | Forward primer | CTCGTGGGTTTGCAGTGAAATTT |
|  | Reverse primer | CCCGATCACTGAACAAGAAAGAA |
| Human CCND1 | Forward primer | CCTCGGTGTCCTACTTCAAATGT |
|  | Reverse primer | TTCATCTTAGAGGCCACGAACAT |
| Human CDK6 | Forward primer | TGACCAGCAGCGGACAAAT |
|  | Reverse primer | GGACTGGAGCAAGACTTCGG |
| Human CHUK | Forward primer | ACCAGCCTCTCAATGTGTTCTAG |
|  | Reverse primer | CCCACACTTTACGCAGCTGTATA |
| Human CXCR4 | Forward primer | TGTCTTGGAGCGAGTTACATTGT |
|  | Reverse primer | GTAGATGGTGGGCAGGAAGATTT |
| Human EGFR | Forward primer | CTGGGTGCGGAAGAGAAAGAATA |
|  | Reverse primer | CCAAAGGTCATCAACTCCCAAAC |
| Human EP300 | Forward primer | CCACCCAACCAGAGGAGAGTATA |
|  | Reverse primer | CAGGAGGATGGCAATGGAAGATA |
| Human ERBB3 | Forward primer | TTGCCATCTTCGTCATGTTGAAC |
|  | Reverse primer | GCCATTGTCCTTCACCACTATCT |
| Human HDAC2 | Forward primer | TGGTGATAGACTGGGTTGTTTCA |
|  | Reverse primer | TGGTGATAGACTGGGTTGTTTCA |
| Human IDH1 | Forward primer | TGTGGTAGAGATGCAAGGAGATG |
|  | Reverse primer | CTCCTCAACCCTCTTCTCATCAG |
| Human IGF1R | Forward primer | ATGCGTGAGAGGATTGAGTTTCT |
|  | Reverse primer | TGGAGGTGCTAGGACTGGATTAT |
| Human MDM2 | Forward primer | CCGGATCTTGATGCTGGTGTATA |
|  | Reverse primer | CCAGTTTGGCTTTCTCAGAGATT |
| Human NFE2L2 | Forward primer | AGCAAGTTTGGGAGGAGCTATTA |
|  | Reverse primer | GAGAGGATGCTGCTGAAGGAATC |
| Human PARP1 | Forward primer | CCAGTTCAGGACCTCATCAAGAT |
|  | Reverse primer | AAGCGATTTGAGAGATCCAGGAT |
| Human PDGFRA | Forward primer | TGTCCTGGTTGTCATTTGGAAAC |
|  | Reverse primer | GCTGCATCGGGTCCACATAAATA |
| Human PTPN11 | Forward primer | CCGCTCATGACTATACGCTAAGA |
|  | Reverse primer | TCCATGATGCTCTCCTGCTTATG |
| Human RPS6KA3 | Forward primer | GCAAGAGAGACCCAACAGAAGAA |
|  | Reverse primer | TCCAGCAATTCACCTCCTTTCAT |
| Human XIAP | Forward primer | TTGGAAGCCCAGTGAAGACC |
|  | Reverse primer | TGTCCTTGAAACTGAACCCCA |
